# Microbial communities in carbonate precipitates from drip waters in Nerja Cave, Spain

**DOI:** 10.7717/peerj.13399

**Published:** 2022-05-03

**Authors:** Valme Jurado, Yolanda Del Rosal, Concepcion Jimenez de Cisneros, Cristina Liñan, Tamara Martin-Pozas, Jose Luis Gonzalez-Pimentel, Bernardo Hermosin, Cesareo Saiz-Jimenez

**Affiliations:** 1 Instituto de Recursos Naturales y Agrobiologia (IRNAS-CSIC), Sevilla, Spain; 2 Instituto de Investigacion Cueva de Nerja, Nerja, Spain; 3 Instituto Andaluz de Ciencias de la Tierra (CSIC-UGR), Armilla, Spain; 4 Departamento de Ecologia y Geologia, Facultad de Ciencias, Universidad de Malaga, Malaga, Spain; 5 Museo Nacional de Ciencias Naturales, Madrid, Spain; 6 Laboratorio Hercules, Universidade de Evora, Evora, Portugal

**Keywords:** Nerja Cave, Carbonate precipitate, Drip water, Bacteria

## Abstract

Research on cave microorganisms has mainly focused on the microbial communities thriving on speleothems, rocks and sediments; however, drip water bacteria and calcite precipitation has received less attention. In this study, microbial communities of carbonate precipitates from drip waters in Nerja, a show cave close to the sea in southeastern Spain, were investigated. We observed a pronounced difference in the bacterial composition of the precipitates, depending on the galleries and halls. The most abundant phylum in the precipitates of the halls close to the cave entrance was *Proteobacteria*, due to the low depth of this sector, the direct influence of a garden on the top soil and the infiltration of waters into the cave, as well as the abundance of members of the order *Hyphomicrobiales*, dispersing from plant roots, and other *Betaproteobacteria* and *Gammaproteobacteria*, common soil inhabitants. The influence of marine aerosols explained the presence of *Marinobacter, Idiomarina, Thalassobaculum, Altererythrobacter* and other bacteria due to the short distance from the cave to the sea. Nineteen out of forty six genera identified in the cave have been reported to precipitate carbonate and likely have a role in mineral deposition.

## Introduction

In karst systems, meteoric waters percolates through rocks reaching caves where it contributes to the dissolution of carbonate rocks and the formation of speleothems as a result of water degassing and evaporation. Speleothems adopt different forms according to the factors involved in its formation: cave location, water flow, organic matter, microbial communities, *etc*. Although most speleothems were usually made of calcium carbonate (Fairchild et al., 2007), siliceous speleothems were reported in volcanic caves (Miller et al., 2015) and in both cases were described the contribution of microorganisms to speleothems formation (De los Ríos et al., 2011; Miller et al., 2020a). Sauro et al. (2018) reported that in orthoquartzite caves, quartz weathering and silica mobility were affected by chemotrophic bacterial communities.

Many hydrological, geochemical and paleoclimate studies have been conducted on speleothems (*e.g.*, Fairchild et al., 2007; Cuthbert et al., 2014; Ellis et al., 2020; Miller et al., 2020a; Miller et al., 2020b and references therein); however the microbiology of drip waters and its contribution to calcite precipitation and speleothem formation is less studied.

A few papers have described the role of bacteria and fungi in speleogenesis. Barton & Northup (2007) reviewed the past, current and future perspectives of cave geomicrobiology and stated that early in the 1960s a few authors suggested that microorganisms played an important role in cave deposits. Further evidences has accumulated across the following decades (Cañaveras et al., 1999; Sanchez-Moral et al., 2003; Cuezva et al., 2012; Maciejewska et al., 2017). It is also probable that drip water bacteria could play a role in cave carbonate deposits.

Microbially induced calcite precipitation (MICP) has been defined as the formation of carbonate minerals from a solution due to the presence of cells, microbial products or metabolic activity (Bosak, 2011). The precipitation can mainly be due to modulation of the environmental pH, nucleation sites on cell surfaces, or by the action of enzymatically driven processes involving carbonic anhydrase, urease, *etc.* (Achal & Pan, 2011; Hoffmann, Reeksting & Gebhard, 2021). Cañaveras et al. (2006) showed that moonmilk consisted of a network of calcite crystals and active filamentous bacteria and concluded that microbes influenced the physico-chemistry of calcite precipitation. Banks et al. (2010) proposed that the formation of speleothems in caves could involve microorganisms active in MICP. They tested the ability of cave bacteria to dissolve and precipitate carbonates and suggested that calcification required a metabolic activity because dead cells were unable to precipitate the minerals.

Cuezva et al. (2012) reported that *Acidobacteria* were able to capture CO_2_ from the air and form calcium carbonate polymorphs. Later, Cuezva et al. (2020) linked the genus *Crossiella* to the ability to capture CO_2_ from the cave atmosphere and precipitate calcium carbonate. Maciejewska et al. (2017) revealed that cave *Streptomyces* were involved in peptides/amino acids ammonification and ureolysis, which increase the pH of the bacterial environment and resulted in carbonate precipitation. Previously, Cañaveras et al. (1999) and Groth et al. (2001) reported the ability of cave *Streptomyces* to precipitate carbonates.

In Nerja Cave, southern Spain, karst hydrodynamic, isotopic and hydrochemical characterization and total organic carbon of drip waters were previously investigated (Liñán et al., 2002; Liñán et al., 2021; Batiot et al., 2003). Jiménez de Cisneros et al. (2020) studied drip water and carbonate precipitates in different halls and galleries of Nerja Cave. They found the presence of microorganisms forming small colonies in some precipitates, which denoted biological activity, but no identification of bacteria was carried out.

In this study we focused our attention on the microbial communities present in the carbonate precipitates along the different galleries and halls. Our aims are to determine the bacteria associated with carbonate precipitation and their source in order to determine the influence of the top soil on meteoric water reaching the cave.

## Materials & Methods

### Geological context and research background

Site description, geological setting, cave microclimatology and hydrology were described elsewhere (Liñán et al., 2002; Liñán et al., 2021; Batiot et al., 2003; Jurado et al., 2020a; Jurado et al., 2021).

### Sampling

**Figure 1:**
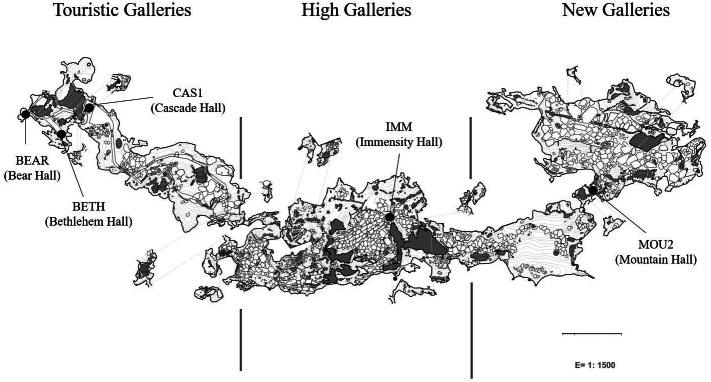
Map of Nerja Cave and location of sampling sites.

Petri dishes were placed across the galleries and halls for eight months (from October 2019 to June 2020) for collecting drip waters and carbonate precipitates. Jiménez de Cisneros et al. (2020) studied the isotopic data and mineralogy of the precipitates collected in the same sites than in this work and identified calcite in the precipitates from Touristic Galleries and calcite and aragonite in the High and New Galleries. Here, we added a new site, Bear Hall and studied the microbial communities of precipitates in five samples (Fig. 1). Bedrock thickness above the sampling points varies depending of the site: 5–8 m in the Touristic Galleries, and about 60–90 m in the High and New Galleries.

### Precipitate characterization

The precipitates were identified by X-ray diffractometry (XRD) using a PANalytical X’PERT PRO diffractometer (PW3071) operating at 45 kV and 40 mA, and employing monochromatic Cu-K *α* radiation at the Institute of Earth Sciences (IACT-CSIC). The XRD spectra were obtained from 10° to 60° 2θ using X’PerHigh Score (PANalytical) software. Samples were examined in a high-resolution scanning electron microscope (HR-SEM) AURIGA from Carl Zeiss, Germany, in the Center for Scientific Instrumentation (CIC, University of Granada).

### Drip water

Periodically, at least once a month, the pH and the electrical conductivity (EC) of the drip points associated to the Petri dishes were measured *in situ*, using HORIBA portable equipment, which allowed taking measurements with a minimum sample volume (drops). pH was measured using a LAQUAtwin pH-11 pH meter (resolution: 0.1 pH, accuracy: ±0.1 pH); EC was measured with the LAQUAtwin B-771 conductivity meter (resolution: 1 µS/cm for conductivity range 9–2,000 µS/cm, accuracy ±2%). For the measure of the flow rate of the drip points a graduated cylinder was used except in Immensity Hall, in which the flow rate was measured by counting the number of drops fallen in 2 min due to its slow flow (Tables S1–S3).

### DNA extraction, sequencing and phylogenetic analysis

The analytical protocols were described elsewhere by Gonzalez-Pimentel et al. (2021). Briefly, genomic DNA were extracted from carbonate precipitates using FastPrep matrix-E lysis tubes (Qbiogene Inc., Carlsbad, CA, USA) with glass beads and physically disrupted in a shaker (Fast Prep-24, Solon, OH, USA). The quality and concentration of the nucleic acids was measured by fluorometric quantification using the Qubit 2.0 fluorometer (Invitrogen, Carlsbad, CA, USA). Samples containing enough DNA for analyses were from Bear Hall (BEAR): 10.5 ng/µL, Bethlehem Hall (BETH): 4.4 ng/µL, and Cascade Hall (CAS1): 4.4 (ng/µL) which appeared in the outermost part of the cave, coinciding with irrigation water locations (Fig. 1). Deeper into the cave, when the thickness of the ceiling progressively increases, DNA concentration decreased to minimal values, likely due to the fact that the water were filtered by the rock. This was not applicable to Immensity Hall (IMM): 4.1 ng/µL and Mountain Hall (MOU2): 0.6 ng/µL, located in the deeper part of the cave. Both samples corresponded to halls in which were suspected a connection with the exterior, based on geophysical and environmental data (Liñán et al., 2021).

Sequencing was performed using Next Generation Sequencing (NGS) of the V3 and V4 regions of the 16S rRNA gene (Takahashi et al., 2014). Library construction was performed using the 16S Metagenomic Sequencing Library preparation protocol (15044223 Rev.B). The generated DNA fragments (DNA libraries) were sequenced with the MiSeq V3 kit from the Illumina MiSeq platform using 300 base pair paired-end reads.

The samples generating from 138,268 (Cascade Hall) to 174,756 reads (Bethlehem Hall) were analyzed, eliminating sequences of low quality, small size and chimeras. Quality control and trimming of raw data were processed using FastQC software and QIIME2 (Bolyen et al., 2019). The elimination of primers, filtering of sequences according to their quality and the grouping of reads in amplicon sequence variants (ASV) were carried out with the DADA2 package (Callahan et al., 2016). 16S copy number corrections and primer biases may limit the data. Taxonomic assignments were based on SILVA database for prokaryotes (version 132) (Quast et al., 2013) for taxonomic identification of 16S rRNA gene sequences (threshold of 80%), and heat-maps built in R using gplots package (Warnes et al., 2016). The functionality of the microbial community in the samples was predicted using FAPROTAX (Louca, Parfrey & Doebeli, 2016). Raw data from 16S-18S rRNA metabarcoding are available at https://www.ncbi.nlm.nih.gov/sra/PRJNA798270.

## Results

### Composition of the microbial communities in carbonate precipitates of Nerja Cave

Our results show that the microbial communities of the five samples were almost entirely composed by *Bacteria*, with percentages ranging between 99.98% and 100% (Table 1). Members of the *Archaea* domain were practically non-existent, reaching 0.01% in Cascade Hall and 0.02% in Bear Hall, both in the Touristic Galleries, but absent in Bethlehem Hall from the same galleries. Other two samples corresponded to the High Galleries (Immensity Hall) and New Galleries (Mountain Hall) where *Archaea* were not detected.

Venn diagram shows amplicon sequence variant (ASV) distribution of prokaryotes in the five samples (Fig. S1). A total of 1,161 ASVs were observed, of which 807 ASVs were unique to drip waters. Most of these unique taxa were found in Bear Hall (315), followed by Mountain Hall (168) and Cascade Hall (121). The common microbial core only comprised 6 distinct ASVs, denoting the extreme diversity of the communities from the waters collected in each hall. The highest number of ASVs was shared between the precipitates from Bethlehem and Bear Halls (35) and Bethlehem and Cascade Halls (25), while Mountain and Bethlehem Halls shared five. These data show the disparity in sample composition.

### Archaeal Phyla

*Archaea*’s phyla distribution was variable, as shown in Table S4. In the precipitates collected in the Touristic Galleries appeared different phyla (*Aenigmarchaeota* in Cascade Hall and *Nanoarchaeota* in the Bear Hall), while unassigned *Archaea* were found in Bear Hall, all of them with abundances of 0.01%.

### Bacterial Phyla

**Figure 2:**
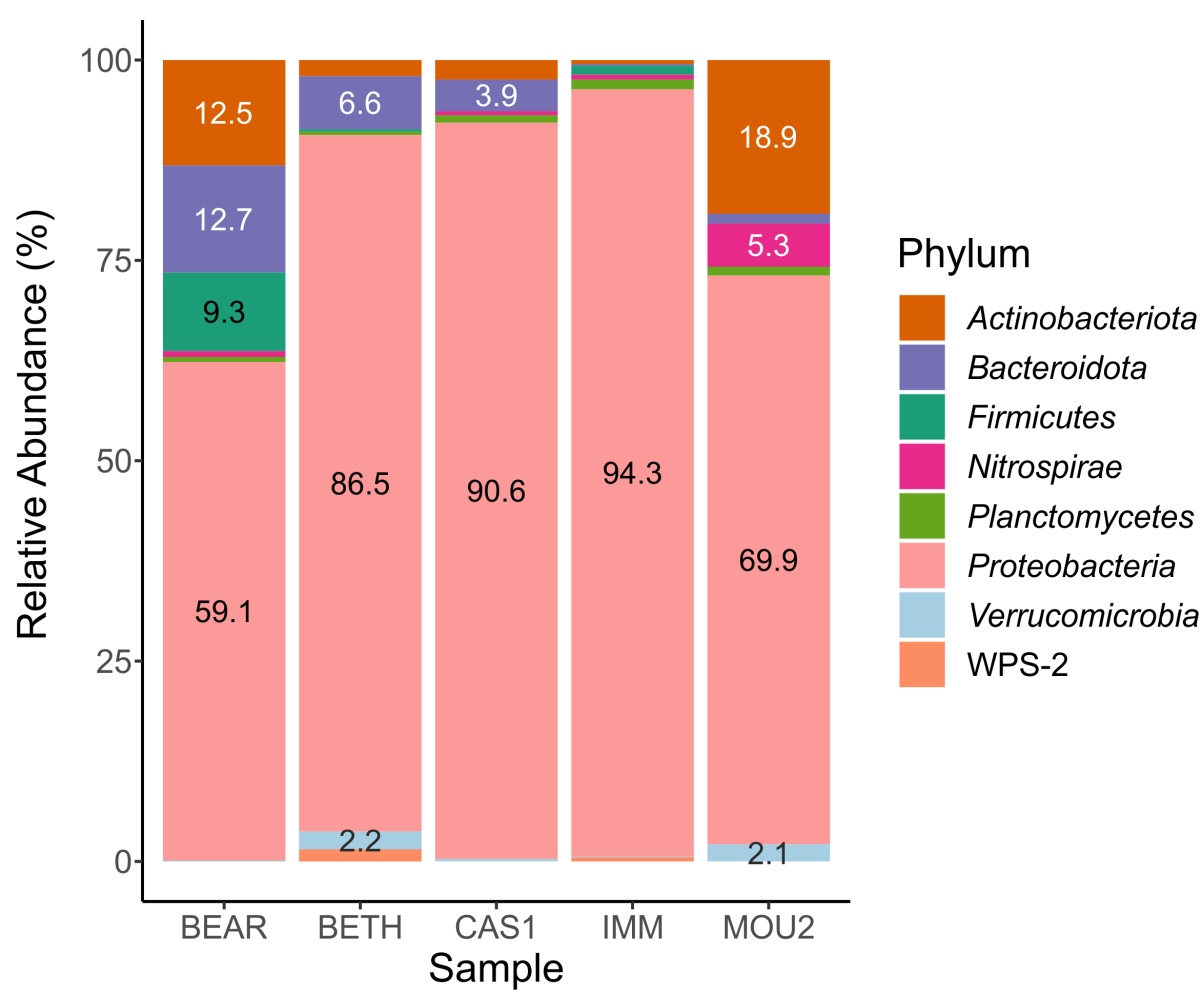
Barplot of bacterial phyla in drip water precipitates from Nerja Cave.

In the *Bacteria* domain, the microbial communities of the drip water precipitates were composed of eight phyla with percentages greater than 1% (Fig. 2). Taxa with abundances less than 1% are not shown in the table.

The most abundant phylum by far was *Proteobacteria*, which varies between 94.3% in Immensity Hall to 59.1% in Bear Hall. Other abundant phyla, between 10 and 20%, were *Actinobacteriota* (Mountain Hall, 18.9% and Bear Hall, 12.5%) and *Bacteroidota* (Bear Hall, 12.7%).

With variable abundances between 5 and 10% appeared *Bacteroidota* (6.6% in Bethlehem Hall), *Firmicutes* (9.3% in Bear Hall) and *Nitrospirae* (5.3% in Mountain Hall). The abundance of the remaining phyla: *Planctomycetes*, *Verrucomicrobia* and WPS-2 (*Candidatus* Eremiobacterota) ranged between 1 and 2%.

**Table 1:** Domains distribution in drip water precipitates from Nerja Cave.

**Domains**	**BETH**	**CAS1**	**BEAR**	**IMM**	**MOU2**
*Bacteria*	100.00	99.99	99.98	100.00	100.00
*Archaea*	0.00	0.01	0.02	0.00	0.00

The almost exclusive abundance of *Proteobacteria* in the precipitates of three Nerja halls (Bethlehem, Cascade, and Immensity) is remarkable, while in another two halls was shared with *Actinobacteriota* and *Bacteroidota* (Bear Hall) or *Actinobacteriota* (Mountain Hall).

**Figure 3:**
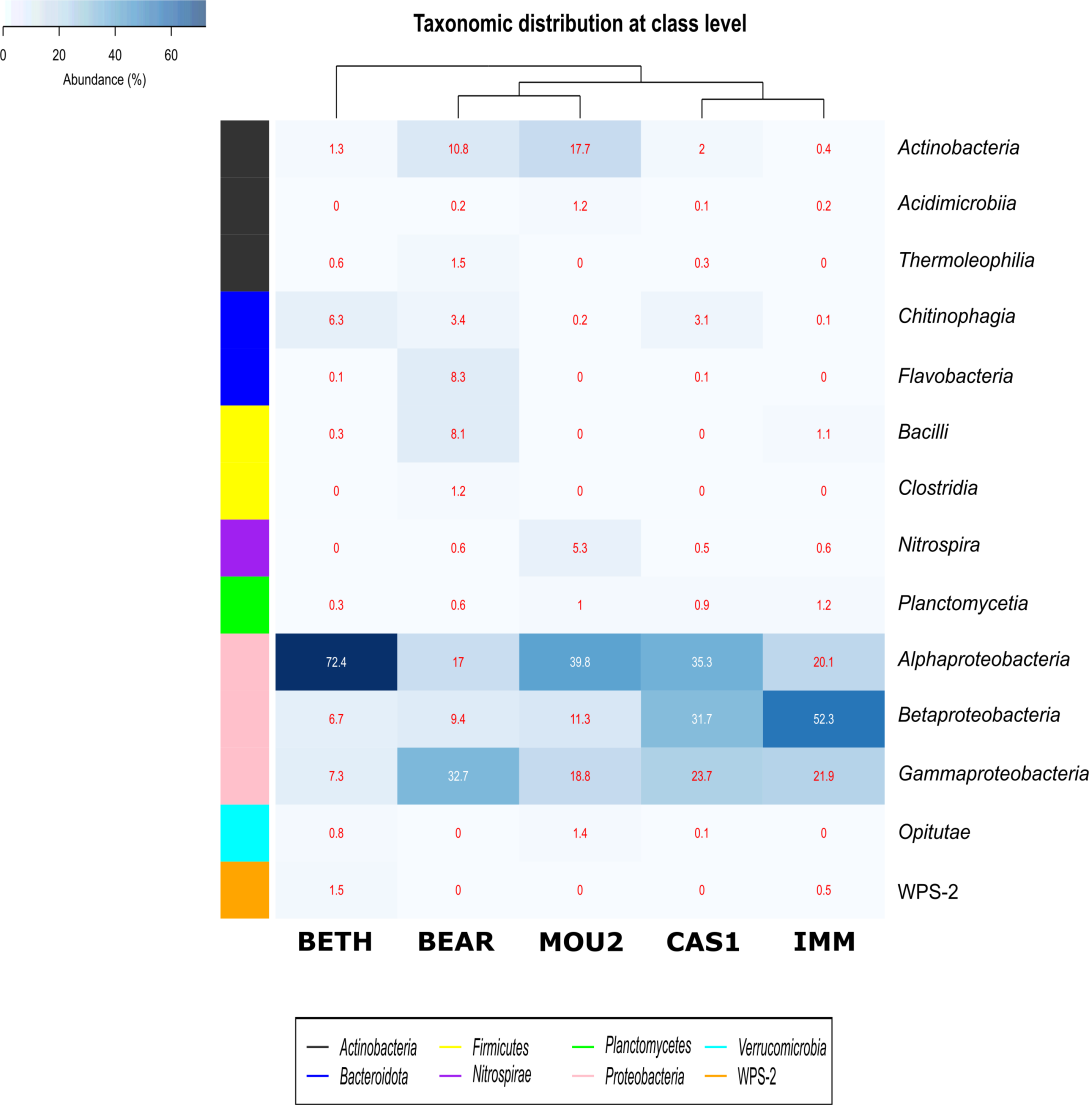
Heat-map of drip water precipitates from Nerja Cave. Taxonomic identifications of *Bacteria* at class level. Classes described in the right column and their abundances included in the boxes. The colored left bar groups the classification at phylum level.

Bacterial classes with a relative abundance >1% in at least one of the precipitates are depicted in Fig. 3. The distribution of *Bacteria* by classes showed that *Proteobacteria* dominated in Nerja precipitates with the classes *Alphaproteobacteria, Betaproteobacteria* and *Gammaproteobacteria*. *Alphaproteobacteria* reached 72.4% in Bethlehem Hall, 39.8% in Mountain Hall, 35.3% in Cascade Hall, 20.1% in Immensity Hall and finally 17.0% in the Bear Hall. *Betaproteobacteria* were well represented in the Immensity Hall (52.3%) and Cascade Hall (31.7%), and with less abundance in Mountain Hall (11.3%), Bear Hall (9.4%) and Bethlehem Hall (6.7%). *Gammaproteobacteria* presented a relative abundance of 32.7% in Bear Hall, followed by 23.7% in Cascade Hall, 21.9% in Immensity Hall, 18.8 in Mountain Hall and 7.3% in Bethlehem Hall.

The majority of *Actinobacteriota* were included in the *Actinobacteria* class (10.8% in Bear Hall and 17.7% in Mountain Hall). Lower percentages were obtained for *Acidimicrobiia* and *Thermoleophilia* with 1.2% in Mountain Hall and 1.5% in Bear Hall and less than 1% in other halls.

In the phylum *Bacteroidota*, two classes *Chitinophagia* and *Flavobacteria* appeared well represented in Bethlehem Hall (6.3%) and Bear Hall (8.3%), respectively, while *Firmicutes* with the class *Bacilli* reached 8.1% in Bear Hall and 1.1% in Immensity Halls. *Clostridia* were only identified in Bear Hall (1.2%). The *Nitrospira* class, phylum *Nitrospirae*, attained 5.3% in Mountain Hall, while in other halls did not exceed 1%.

*Plantomycetia* was present in all the samples, but only in two halls showed relatives abundances > 1%: 1.2% in Immensity Hall and 1% in Mountain Hall.

The class *Opitutae* appeared with low relative abundances in three halls (Bethlehem, Mountain, and Cascade Halls), as well as the class WPS-2, recently proposed as *Candidatus* Eremiobacterota (Ji et al., 2021), only present in Bethlehem and Immensity halls.

**Figure 4:**
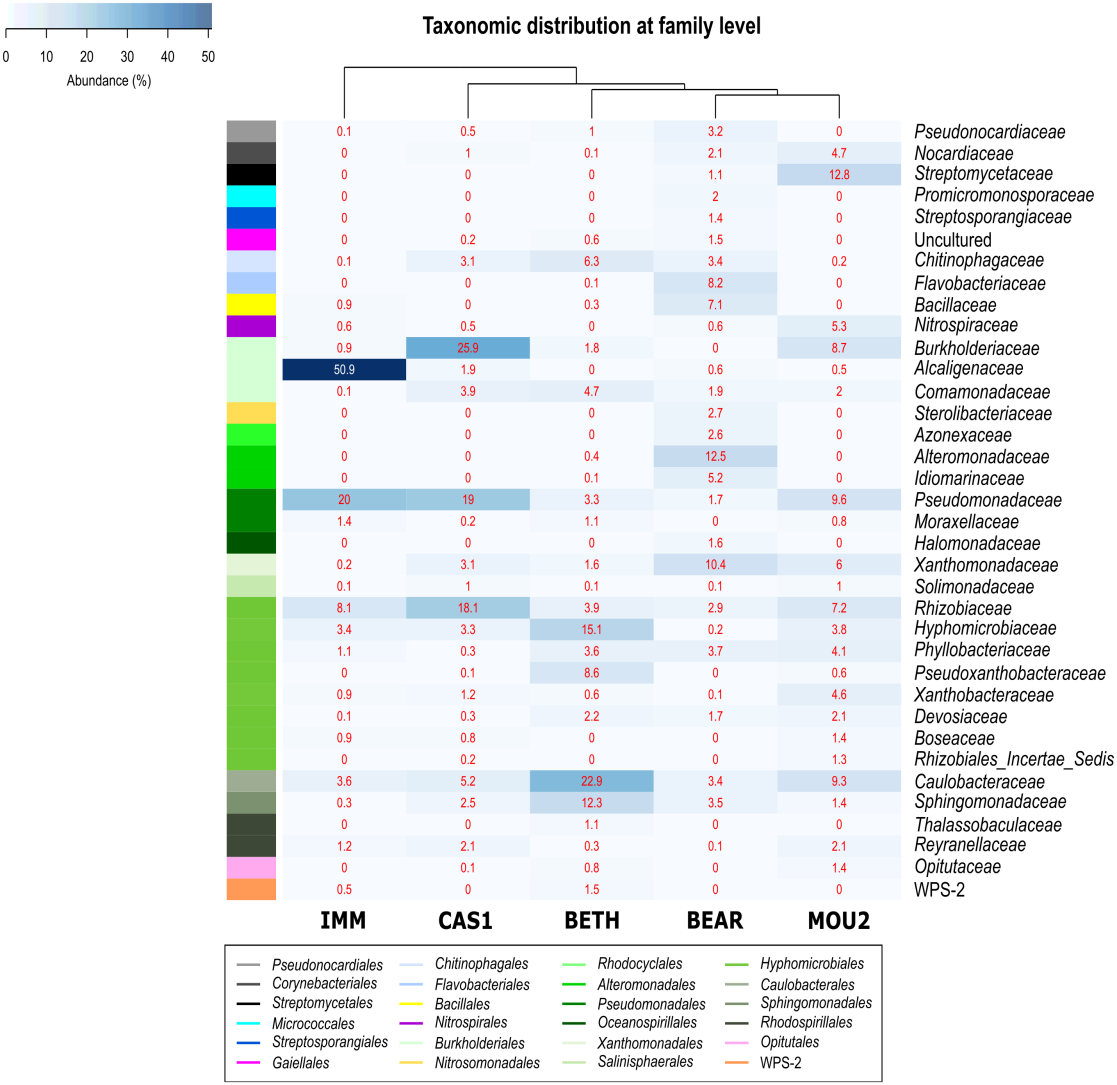
Heat-map of drip water precipitates from Nerja Cave. Taxonomic identifications of *Bacteria* at family level. Families described in the right column and their abundances included in the boxes. The colored left bar groups the classification at order level.

Ten families dominate Nerja precipitates with relative abundances over 10%: *Streptomycetaceae, Burkholderiaceae, Alcaligenaceae, Alteromonadaceae, Pseudomonadaceae, Xanthomonadaceae, Rhizobiaceae, Hyphomicrobiaceae, Caulobacteraceae* and *Sphingomonadaceae* (Fig. 4). They showed the highest relative abundances in the Immensity, Cascade and Bethlehem halls, where was noticed 50.9% of *Alcaligenaceae* and 20.0% of *Pseudomonadaceae* in the Immensity Hall; 25.9% for *Burkholderiaceae*, 19.0% for *Pseudomonadaceae* and 18.1% for *Rhizobiaceae* in Cascade Hall; and 15.1% for *Hyphomicrobiaceae,* 22.9% for *Caulobacterraceae* and 12.3% for *Sphingomonadaceae* in Bethlehem Hall.

In Bear Hall only *Alteromonadaceae* (12.5%) and *Xanthomonadaceae* (10.4%) and in Mountain Hall *Streptomycetaceae* (12.8%) showed relative abundances > 10%.

**Figure 5:**
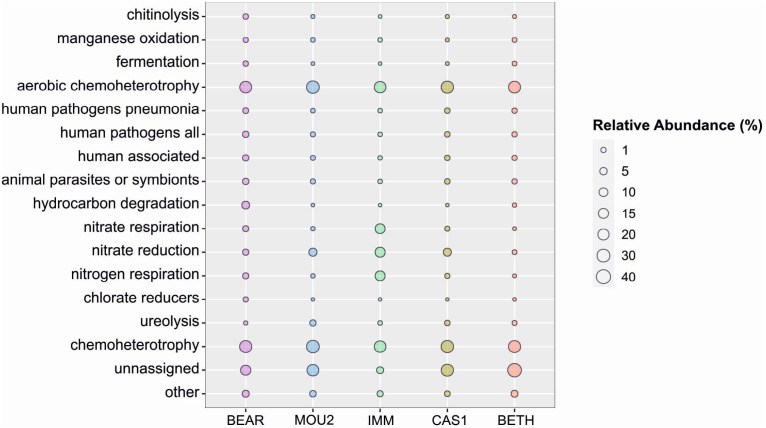
Predicted ecological functions in Nerja Cave drip water precipitates. Circle size indicates relative abundance.

The prediction of ecological functions using FAPROTAX tool is shown in Fig. 5. The most abundant function of the microbial community in the carbonate precipitates is heterotrophism, both under aerobic and anaerobic conditions. This is consistent with the high number of members of the *Proteobacteria* and *Bacteroidota*, two well-known copiotrophic phyla. This is followed by a group of unassigned functions, due to the lack of information in the database. Thirdly, it highlights the bacteria involved in the nitrogen cycle, particularly abundant in the precipitates of the Immensity Hall (respiration of nitrogen and nitrate, and reduction of nitrate), although the reduction of nitrogen is higher in Cascade and Mountain Halls with respect to the two other halls.

### Calcite precipitates

**Figure 6:**
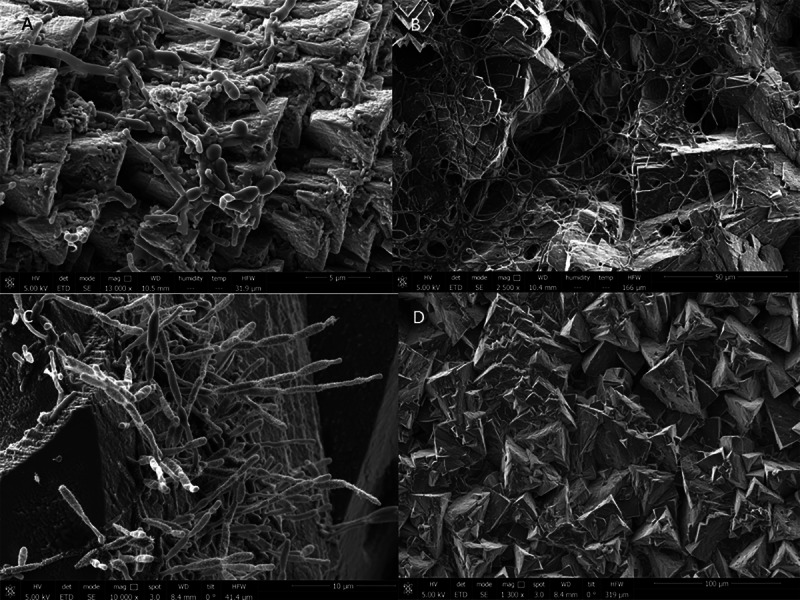
High-resolution scanning electron microscopy of precipitates from Nerja Cave drip waters and biofilms. (A) Bear Hall. (B) Bethlehem Hall. (C) Cascade Hall. (D) Mountain Hall.

The mineralogical study revealed a calcium carbonate composition of the precipitates, appearing the two polymorphic phases of aragonite and calcite (Fig. S2). The precipitates obtained mainly responded to equidimensional crystals of calcite, with a homogeneous growth in all directions. These crystals of rhombohedral habit constituted a true “mesocrystals assembly”. The precipitates of the High and New Galleries generally showed no signs of dissolution and also corresponded to well-formed calcite crystals with rhombohedral habits, with scarce presence of microbial cells (Fig. 6D). In these galleries were also detected crystals with a tabular habit that to corresponded to aragonite phases. However, in the Touristic Galleries, the abundant presence of microorganisms forming biofilms was observed, as shown in Figs. 6A–6C. The biofilms can be related to some defects that appeared in the crystals, such as perforations, which were very abundant in the precipitates of some of the galleries causing the alteration of its structure and morphology.

## Discussion

### Bacterial genera in carbonate precipitates

The sparse distribution of *Archaea* in precipitates is common in caves, where they do not seem to play a significant role in the composition of the community (Jurado et al., 2020a; Jurado et al., 2020b; Addesso et al., 2021; Gonzalez-Pimentel et al., 2021).

*Aenigmarchaeota* is a candidate phylum whose members were initially described in the waters of a deep gold mine and later found in soils and sediments of a hot spring (Rinke et al., 2013). Although there are few genomes available for study, it has been proposed that members of the phylum *Aenigmarchaeota*, due to their limited metabolic capacities, are symbionts of other bacteria. The phylum *Nanoarchaeota* is composed of obligate symbionts with other *Archaea* in hydrothermal environments (Clingenpeel et al., 2013) and in the precipitates is represented by the order *Woesearchaeales* found in Bear Hall.

Both *Aenigmarchaeota* and *Nanoarchaeota* were detected in vermiculations, mineral deposits on rock surfaces that can be found in caves all over the world (Jurado et al., 2020b; Addesso et al., 2021). Their occurrence in the precipitates is possibly related to transport by drip waters, as they were only present in the halls with least bedrock thickness.

Within the *Bacteria* the phyla *Proteobacteria* and *Actinobacteriota* and many of their genera were abundant in the precipitates, which is consistent with previous data from soils and caves (Delgado-Baquerizo et al., 2018; Jurado et al., 2020a; Jurado et al., 2020b; Farda et al., 2022). However, the low relevance of *Firmicutes* is in accordance with the nature of the samples. *Firmicutes* were abundantly represented in Spanish caves, both in air and sediments because their members form spores under adverse conditions (Dominguez-Moñino et al., 2018; Dominguez-Moñino et al., 2021). Laiz et al. (1999) found *Bacillus* in the drip waters of Altamira Cave. *Bacillus* spp. have a remarkable role in calcium carbonate precipitation (Naveed et al., 2020; Rajasekar et al., 2021).

Given the number of genera in the precipitates of Nerja Cave (Table S5), those that reached relative abundances greater than 10% in at least one of the samples will be discussed, although due to their ecological features a few more genera with lower abundances will also be considered. These genera can be distributed in three different groups according to their ecology: soil and cave, aquatic, and predatory bacteria.

With relative abundances over 10% we found seven genera (*Achromobacter, Brevundimonas, Pseudomonas, Hyphomicrobium, Streptomyces, Sphingopyxis* and *Ensifer*) which are common cave inhabitants. All except *Achromobacter* and *Sphingopyxis* were included in the global atlas of dominant soil bacteria (Delgado-Baquerizo et al., 2018). A search in genome databases confirmed that most of these genera contain carbonic anhydrase genes that could justify their involvement in the precipitates (Emameh et al., 2018).

*Achromobacter* represented 50.9% of relative abundance in Immensity Hall, while in other halls did not exceed 1.9% (Cascade Hall) or was less than 1%. *Achromobacter* was previously reported in caves (Bastian, Alabouvette & Saiz-Jimenez, 2009; Banerjee & Joshi, 2016; Gan et al., 2019) and an *Achromobacter* strain isolated from a coastal cave precipitate calcium carbonate (Busquets et al., 2014).

*Brevundimonas* was found in Bethlehem Hall (22.3%). *Brevundimonas diminuta* was identified in Cascade Hall and Immensity Hall, while *Brevundimonas abyssalis* was found in Cascade Hall. These two species of *Brevundimonas* were previously detected in other Spanish caves (Dominguez-Moñino et al., 2021), while unidentified species of this genus were reported in phototrophic biofilms developed on Nerja speleothems (Jurado et al., 2020a). It is noteworthy that *B. diminuta* and other *Brevundimonas* spp. can precipitate calcium carbonate (Okyay & Rodrigues, 2015; Ali et al., 2022) which could explain their occurrence in Nerja precipitates.

*Pseudomonas* is a genus widely distributed in soils and caves (Liu et al., 2010; Campbell et al., 2011; Bastian, Alabouvette & Saiz-Jimenez, 2009; Dominguez-Moñino et al., 2021). In Nerja Cave appeared with abundance in Cascade (19.0%) and Immensity halls (20.0%). However, while in the first hall *Pseudomonas alcaligenes* reached an abundance of 12.3%, in the Immensity Hall no known species was identified. Several *Pseudomonas* spp. genomes revealed the presence of carbonic anhydrase genes (Smith & Ferry, 2000; Sharma, Bhattacharya & Singh, 2009; Emameh et al., 2018) involved in calcium biomineralization.

*Streptomyces* is an abundant genus in the air and mineral surfaces of subterranean environments (Niyomvong et al., 2012; Maciejewska et al., 2017; Dominguez-Moñino et al., 2021; Farda et al., 2022) but only reached a representative abundance in Mountain Hall (12.8%) with the species *Streptomyces aculeolatus* (12.4%) originally isolated from soils in Japan (Shomura et al., 1987). No report on the occurrence of *Streptomyces aculeolatus* in caves was found. In Bear Hall *Streptomyces* only attained 1.1%, while it was absent in other halls. Many *Streptomyces* spp. contains carbonic anhydrase genes (Emameh et al., 2018) and have been involved in calcium carbonate precipitation (Cañaveras et al., 1999; Groth et al., 2001).

*Hyphomicrobium* contains denitrifying methylotrophic species which are common in caves (Manolache & Onac, 2000; Northup et al., 2003; Gonzalez-Pimentel et al., 2021; Vaccarelli et al., 2021). This genus was associated with *Caulobacter* and *Pedomicrobium* (Vaccarelli et al., 2021), all of them with the ability to oxidize manganese. Northup et al. (2003) found the presence of iron and manganese oxidizing bacteria, including *Hyphomicrobium, Pedomicrobium, Leptospirillum, Stenotrophomonas* and *Pantoea* in ferromanganese deposits of Lechuguilla and Spider caves. Despite the abundance of *Hyphomicrobium* in Bethlehem Hall, only *Hyphomicrobium vulgare* was identified in the Immensity and Mountain Halls, indicating that likely other unidentified species occurred in the cave.

*Sphingopyxis* was identified in all the samples, with relative abundances ranging between 10.8% in Bethlehem Hall and 0.1% in Immensity Hall. Different species of *Sphingopyxis* were found in contaminated soils (Sharma et al., 2021) and subterranean environments. Gan et al. (2014) studied the genome of two species of *Sphingopyxis*, isolated from Lechuguilla Cave in New Mexico, while unidentified species were found in other caves (Marques et al., 2019). A survey on genome databases revealed that species of *Hyphomicrobium* and *Sphingopyxis* contained carbonic anhydrase, as well as *Ensifer*.

The genus *Ensifer* (= *Sinorhizobium*) comprises nitrogen-fixing symbiotic bacteria. *Ensifer adherens* has predatory activity on other bacteria (Casida, 1982) and was found in caves from India (Banerjee & Joshi, 2016), USA (Kumar et al., 2017) and Galapagos Islands (Miller et al., 2020a). In the precipitates only attained importance in Cascade Hall.

With low relative abundances was retrieved *Nitrospira* that reached its maximum abundance in Mountain Hall (5.3%), while in other halls the percentages were lower than 1%. This genus comprises ammonium oxidizing bacteria and is relatively frequent in caves (Jurado et al., 2020b; Martin-Pozas et al., 2020; Gonzalez-Pimentel et al., 2021). Also involved in the nitrogen cycle is *Mesorhizobium*, a nitrogen-fixing bacterium, found in all precipitates with relative abundances between 0.1% and 3.7% except in Immensity Hall, and previously identified in caves (Wischer et al., 2015).

The family *Rhizobiaceae* (order *Hyphomicrobiales*) is diverse with about 170 validly recognized species distributed in 17 genera, among which are four genera *Allorhizobium, Neorhizobium, Pararhizobium* and *Rhizobium*, which could not be separated in this study. Species belonging to the *Rhizobiaceae* have been found in subsurface environments since they can be transported from root nodules to the cave by meteoric waters or by the roots when penetrating the cave ceiling. Diaz-Herraiz et al. (2014) detected a high abundance of *Hyphomicrobiales* in an Etruscan tomb with many roots hanging from the ceiling.

A few genera: *Limnobacter*, *Marinobacter, Salegentibacter, Idiomarina, Lacunisphaera, Aliihoeflea,* and *Altererythrobacter* are of interest since were common in marine and continental aquatic environments, but only *Altererythrobacter* was previously found in caves (Wischer et al., 2015). Most of these genera reflect the influence of marine aerosols on the cave environment. *Limnobacter* reached an abundance of 25.8% in Cascade Hall and lower in Mountain Hall (8.7%) and Bethlehem Hall (1.8%), being absent in other two halls. *Marinobacter* is one of the dominant genera in marine environments (Ng et al., 2014), and rarely found in terrestrial environments (Nie et al., 2021). In Nerja only appeared in Bear Hall (12.5%) and Bethlehem Hall (0.4%).

The remaining genera reached relative abundances from 6.0 to 0.1% in the halls of the Touristic Galleries, the ones closest to the sea. Most species of *Salegentibacter* were isolated from marine sediments (Lian et al., 2021). Members of the genus *Idiomarina* have a defined ecological behavior since they need sodium chloride for their growth and were found in salterns and in the oceans (Albuquerque & Da Costa, 2014). The genus *Lacunisphaera* was described from three new species isolated from a lake and was characterized by the fact that their cell walls have peptidoglycan, unlike other members of the *Verrucomicrobia* (Rast et al., 2017). This genus was one of the most abundant in wetlands with a marked role in the dissimilatory reduction of nitrate (Zhao et al., 2020). The genus *Aliihoeflea*, proposed by Roh et al. (2008) and not validated by the scientific community, was found in Bethlehem, Cascade and Bear halls. Another genus whose species were isolated from seawater is *Altererythrobacter* (Yang et al., 2014). Wischer et al. (2015) reported the methylotrophic activity of *Altererythrobacter* in Movile Cave, Romania.

In the group of predatory and/or bacteria associated to amoebae we included *Stenotrophomonas maltophilia* (Denet et al., 2018). This species is relatively common in caves (Bastian, Alabouvette & Saiz-Jimenez, 2009; Urzì et al., 2010). Other genera related to amoebas were *Reyranella* and *Bosea* (La Scola et al., 2003; Pagnier, Raoult & La Scola, 2011). The presence of *Stenotrophomonas*, *Reyranella*, and *Bosea* points to a likely association with amoebae in the drip waters of Nerja Cave. Previously, abundant *Amoebozoa* were found on the phototrophic biofilms of this cave (Jurado et al., 2020a).

*Lysobacter* comprises species predating on other Gram positive and negative bacteria, filamentous fungi, cyanobacteria, algae and nematodes (Reichenbach, 2006) and are frequently isolated from soils and caves (Chen et al., 2016; Kim et al., 2019). In Nerja Cave, *Lysobacter* appeared with significant abundance in areas from a speleothem where the biofilms had disappeared, which led to the conclusion that this genus could play a role in the control of phototrophic communities (Jurado et al., 2020a).

### Prediction of ecological functions

The predicted ecological functions of the bacterial communities presented a high proportion of sequences assigned to chemoheterotrophy.

Several *Achromobacter* species (Doi et al., 2014) and *Dechloromonas* (Duffner et al., 2021) present in the precipitates of Immensity Hall, and *Stenotrophomonas nitritireducens* (Finkmann et al., 2000) in Bear Hall were associated with nitrogen metabolism.

In terms of nitrate reduction, *Streptomyces aculeolatus* found in the Mountain Hall stands out with *Ensifer* in Cascade Hall, *Achromobacter* in Immensity Hall and *Dechloromonas* in Bear Hall in smaller proportions. Species of *Streptomyces* are also through to be involved in nitrate reduction (Feng et al., 2014; Fischer et al., 2014), as well as *Ensifer* (Torres et al., 2014).

Sequences assigned to hydrocarbon degradation occurs in the Bear Hall highlighted by *Marinobacter* and *Halomonas* (Gauthier et al., 1992; Gasperotti et al., 2015). In Mountain Hall the participation of *Mesorhizobium* in ureolysis was significant. However, all samples showed evidence of ureolysis, a process that involves calcium carbonate bioprecipitation (Omoregie, Palombo & Nissom, 2021).

### Bacteria involved in calcite precipitation

The bacteria found in the precipitates of the different halls can have different origins: (i) from drip waters, or (ii) transported by air and deposited on the plates. Based on this, it seems appropriate to carry out a search for the genera (>1% relative abundance) found in the five samples and their possible role in calcite precipitation.

The precipitates from the five halls show a considerable abundance of bacterial genera with the ability to induce calcite precipitation. Table S6 shows 19 genera of bacteria found in the precipitates of the cave drip waters with species that have been described as inducing calcite precipitation. This represented 41.3% of the bacteria identified in Nerja Cave.

For example, in Bear Hall were identified *Marinobacter* (12.5%), *Idiomarina* (5.2%), *Bacillus* (3.1%), *Lysobacter* (3.1%) and *Stenotrophomonas* (2.1%), which represented 26% of the total abundance; in Bethlehem Hall *Brevundimonas* (22.3%) and *Sphingopyxis* (10.8%) amounted 33.1%; in Cascade Hall *Pseudomonas* (19.0%), *Ensifer* (10.8%) and *Caulobacter* (4.5%) attained 34.3%; in Immensity Hall *Achromobacter* (50.9%) and *Pseudomonas* (20.0%) reached 70.9%; and in Mountain Hall *Streptomyces* (12.8%) and *Caulobacter* (8.5%) 21.3%.

These data could support the hypothesis of a biogenic calcite precipitation, without ruling out that abiotic precipitation could also have occurred in parallel due to degassing and evaporation of the drip water. In favor of a biogenic precipitation were the SEM observations (Fig. 6) and previously published data. In fact, Jiménez de Cisneros et al. (2020) observed how the values of *δ*^13^C in the precipitates showed a greater variability that can be explained by the presence of vegetation on the surface and by the residence time of water in the soil. On the other hand, the presence of microorganisms in the precipitates obtained from the Touristic Galleries could explain the more negative *δ*^13^C values obtained in these samples.

There is a great difference in the composition of the microbial communities of the drip water precipitates in Nerja Cave. This can be attributed, without a doubt, to the diverse ecological niches existing along the different cave sectors. Above the Touristic Galleries there is an extensive garden of *Casuarina*, *Cupressaceae* (cypresses), *Arecaceae* (palm trees) and *Pinaceae* (pines) (Docampo et al., 2007). Due to the scarce thickness from the top soil to the cave ceiling (about 5–8 m), the waters infiltrate in favor of fissures and the hollows of roots. On the soils above the other two galleries, the predominant vegetation is natural and adapted to the ecological conditions of a poorly developed calcareous soil (Jiménez de Cisneros et al., 2020), in which the thickness of the rocks ranges between 60–70 m in the High Galleries up to 90 m in the New Galleries.

Drip water organic carbon contents were high in the garden soils near the cave entrance and the Touristic Galleries (7–9%), while in the other two galleries it was three times lower (Jiménez de Cisneros et al., 2020). This obviously has influence both on the soil microbial communities, and on the infiltration waters. In fact, Batiot et al. (2003) reported high concentrations of organic carbon in the drip water of the Touristic Galleries (1 to 5 mg/l, with a mean value of 2.2 mg/l,) and related them to the elevated content of organic matter in the top soils of the studied area.

In Bear Hall *Proteobacteria* reached a relative abundance of 59.1%, followed by *Bacteroidota* (12.7%), *Actinobacteriota* (12.5%) and *Firmicutes* (9.3%). This pattern was similar to those of soil communities, where these four phyla were usually well represented (Patel et al., 2016; Mhete et al., 2020). Mountain Hall deviates from the common pattern of all samples, with the highest abundance of *Actinobacteriota* (genus *Streptomyces*) and *Nitrospirae* (genus *Nitrospira*). *Nitrospira* was found in an extreme environment (Vapor Cave) at depths of up to 80 m (Martin-Pozas et al., 2020). Koch, van Kessel & Lücker (2019) suggested a metabolic versatility, and the adaptation of *Nitrospira*, a nitrifying bacterium, to microaerophilic and oligotrophic conditions (Daims & Wagner, 2018), which would coincide with the rock thickness in this area (90 m). *Streptomyces* is common in the air, rocks and sediments of show caves (Groth et al., 1999; Groth et al., 2001; Dominguez-Moñino et al., 2021). Liñán et al. (2021) suggested, based on ventilation patterns, the connection of Nerja with another cave, which could explain the abundance of *Streptomyces*.

The abundances of *Achromobacter* and *Pseudomonas* in the precipitates from Immensity Hall are noteworthy. This together with the presence of *Hyphomicrobiales* and *Caulobacterales* (Table S5) pointed to the influence of soil bacteria. Previous data suggested a connection of this hall with the exterior (Liñán et al., 2021).

Of the five samples studied, only the corresponding to the Touristic Galleries showed a very low relative abundance of *Archaea*, which can be attributed to their transport from the garden soil to the cave. Indeed, soil microbial communities were usually composed of *Bacteria* that dominate in metagenomes, while *Archaea* were rare (<3%), according to Bates et al. (2011).

What is the contribution of airborne bacteria to the precipitates, due to the plates being exposed to open air for several months? Unfortunately, we do not have data at present on airborne bacteria in Nerja Cave. Previous data showed that *Bacillus* and *Micrococcus* were the most abundant genera in the air of Touristic Galleries (Del Rosal et al., 2007; Del Rosal, Liñán & Hernández-Mariné, 2014). *Bacillus* was identified in the precipitates with low relative abundance, but not *Micrococcus*. Moreover, an aerobiological study on two caves distant 50 km (Tesoro Cave, Rincon de la Victoria) and 100 km (Ardales Cave, Ardales) was very conclusive in this respect. In fact, in both caves the most abundant bacterial genera were *Micrococcus* and *Arthrobacter* as detected along the four seasons and in all the halls. Both genera were also abundant outdoor (Dominguez-Moñino et al., 2021). Interestingly none of these two genera were identified in the precipitates which suggest a scarce or null contribution of airborne bacteria.

Other caves, Gruta de las Maravillas (at 350 km from Nerja) and Altamira (at 940 km from Nerja) also presented a similar pattern with a notable abundance of *Micrococcus* in all the halls and seasons (Garcia-Anton et al., 2014; Dominguez-Moñino et al., 2021).

## Conclusions

The microbial communities from carbonate precipitates in Nerja Cave are quite diverse and reflect the influence of distinct drip waters, depending on the areas where the galleries and halls are located. These drip waters seem to be mainly influenced by top soil inputs. In fact, *Proteobacteria*, the most abundant phylum in the precipitates of drip waters of the Touristic Galleries, are related to the soil due to the low depth of this area and the direct influence of the garden and the infiltration waters. This is also supported by the important number of members of the order *Hyphomicrobiales*, undoubtedly originating from the roots of garden plants, and other *Alphaproteobacteria* and *Gammaproteobacteria*, common soil inhabitants.

The influence of marine aerosols could explain the presence of *Marinobacter, Idiomarina, Thalassobaculum, Altererythrobacter* and other bacteria due to the short distance between the cave and the sea.

The high number of genera related with carbonate precipitation and their abundance on the precipitates, as shown the SEM observations, are highly suggestive of the involvement of bacteria in the process.

##  Supplemental Information

10.7717/peerj.13399/supp-1 Supplemental Information 1 Supplemental Materials

## Additional Information and Declarations

### Data Availability

The following information was supplied regarding data availability: The raw data are available at NCBI SRA PRJNA798270.
