# Supplementary material for: Microbial communities in carbonate precipitates from drip waters in Nerja Cave, Spain
Source: PeerJ. 2022 May 3;10:e13399. doi: 10.7717/peerj.13399 (PMC9074860; doi:10.7717/peerj.13399)
Supplement: Supplemental Information 1 [file peerj-10-13399-s001.docx]

MICROBIAL COMMUNITIES IN CARBONATE PRECIPITATES FROM DRIP WATERS IN NERJA CAVE. SPAIN

Valme Jurado^1^, Yolanda del Rosal^2^, Concepcion Jimenez de Cisneros^3^, Cristina Liñán^2.4^, Tamara Martin-Pozas^5^, Jose Luis Gonzalez-Pimentel^6^, Bernardo Hermosin^1^, Cesareo Saiz-Jimenez^1*^

^1^Instituto de Recursos Naturales y Agrobiologia (IRNAS-CSIC), 41012 Sevilla, Spain

^2^Instituto de Investigacion Cueva de Nerja, 29787 Nerja, Spain

^3^Instituto Andaluz de Ciencias de la Tierra (CSIC-UGR), 18100 Armilla, Spain

^4^Departamento de Ecologia y Geologia, Facultad de Ciencias, Universidad de Málaga, 29071 Malaga, Spain

^5^Museo Nacional de Ciencias Naturales (MNCN-CSIC), 28006 Madrid, Spain

^6^Laboratorio Hercules, Universidade de Evora, 7000-809 Evora, Portugal

*** Correspondence:**Cesareo Saiz-Jimenez
saiz@irnase.csic.es


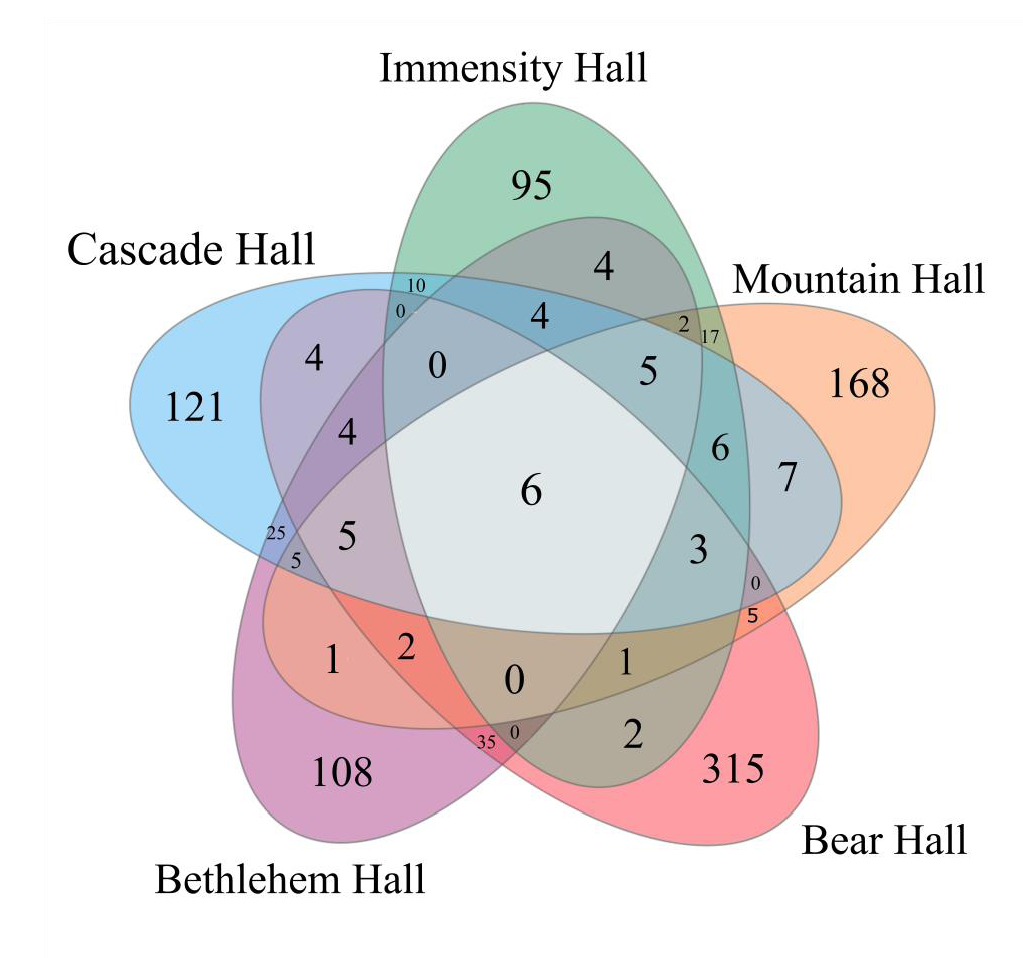


**Figure S1.** Venn diagram of five drip water precipitates from Nerja Cave.


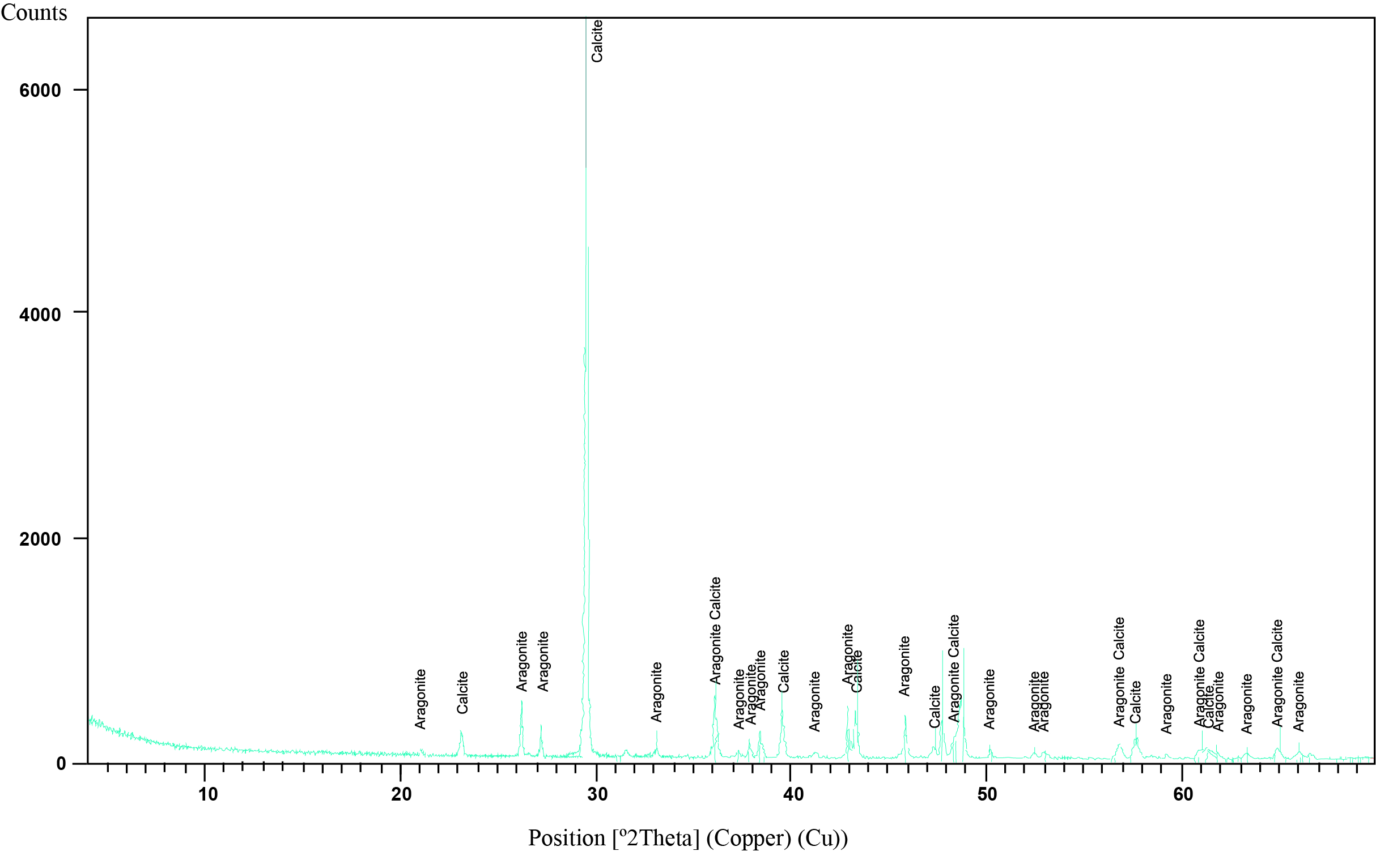


**Figure S2.** Representative powder X-ray diffraction pattern of precipitates in Nerja Cave (sample CAS1, Cascade Hall). The peaks of aragonite and calcite are indexed.

**Drip waters**

The electrical conductivity values of drip waters vary between 410 and 3,600 μS/cm. In the Touristic Galleries (Bear, Bethlehem, and Cascade halls) the drip waters have electrical conductivity values (900 to 3,600 μS/cm), with a mean value of 1,845 μS/cm, higher than in the other two galleries (Table S1). The pH is basic with values comprised between 7.0 and 8.4 and the flow rate (Q) is variable, with mean values between 1.0 and 9.0 ml/min (Table S2). Bear and Bethlehem halls dripping points remain dry for most of the studied period, specially the first. The values measured are therefore similar to the historical data measured in Cascade Hall point from 1991 to 2018: EC: 1,218 μS/cm, pH: 7.33, Q: 10 ml/min (mean values).

The drip waters in Touristic Galleries are HCO_3_-SO_4_ Ca-Mg type (*Carrasco Cantos et al.* *2002*) and its origin is related to the rainwater infiltration but overall, to the irrigation of the gardens located above this cave sector. So, its higher or lower flow rate depends on the frequency of irrigation and the amount of water used for it. This water comes from a nearby well and, occasionally, from Maro spring so they are waters coming from the unsaturated zone of the aquifer*. Carrasco Cantos et al. (2002)* reported in Cascade Hall high concentration in SO_4_^2-^, Cl^-^ and Na^+^, originated from dissolution of these components in the anthropic soil and re-concentration of the rainfall.

The electrical conductivity values of drip waters in the non-visited sector (Immensity and Mountain halls) vary between 410 and 700 μS/cm, with a mean value of 523 μS/cm (Table S1). The pH is basic with values comprised between 7.4 and 8.4 (Table S2). A single flow rate measurement is available for the studied period in these dripping points (Immensity Hall: 1 drop/min, Mountain Hall: 1 ml/min) (Table S3) but previous data (2017-2018) showed that the mean flow rate in Mountain Hall is low, 5 ml/min (*Jiménez de Cisneros et al., 2020*), although flow increases were observed in response to rain episodes (*Liñán et al., 2008*). The historical data of EC and pH measured in MOU2 from 2003 to 2018 (603 μS/cm, 7.80) were similar to those obtained in this study. These waters are Ca-Mg-HCO_3_ type (*Carrasco Cantos et* *al. 2002*) because in this cave sector there is no infiltration of irrigation water, since they are exclusively of meteoric origin.

**Table S1.** Electrical conductivity (in μS/cm) of drip waters of Nerja Cave (period 2019-2020). n: number of samples, max: maximum value, min: minimum value, mean: average value, s: standard deviation, v: variation coefficient.

**Table S2.** pH of drip waters of Nerja Cave (period 2019-2020). n: number of samples, max: maximum value, min: minimum value, mean: average value, s: standard deviation, v: variation coefficient.

**Table S3.** Flow rate for the studied drip water points for the period 2019-2020 in ml/min (BEAR; BETH; CAS1, MOU2) and number of drops/min (IMM). n: number of samples, max: maximum value, min: minimum value, mean: average value, s: standard deviation, v: variation coefficient. Gray boxes: water is observed in the studied drip water points (they aren´t dry although their flow rate is lower than 1 ml/min, the minimum value measurable with the graduated cylinder).

**Table S4.** Archaeal distribution in drip water precipitates from Nerja Cave.

| **Phylum** | **Class** | **Order** | | **Family** | **BETH** | **CAS1** | **BEAR** | **IMM** | **MOU2** |
| --- | --- | --- | --- | --- | --- | --- | --- | --- | --- |
| *Aenigmarchaeota* | Unassigned *Aenigmarchaeota* | | | | 0.00 | 0.01 | 0.00 | 0.00 | 0.00 |
| Unassigned *Archaea* | | | | | 0.00 | 0.00 | 0.01 | 0.00 | 0.00 |
| *Nanoarchaeota* | *Nanoarchaeia* | | *Woesearchaeales* | GW2011_GWC1_47_15 | 0.00 | 0.00 | 0.01 | 0.00 | 0.00 |

**Table S5**. Relative abundance (>1%) of bacterial genera in precipitates of drip waters from Nerja Cave

| **Phylum** | **Class** | **Order** | **Family** | **Genus** | **BETH** | **CAS1** | **BEAR** | **IMM** | **MOU2** |
| --- | --- | --- | --- | --- | --- | --- | --- | --- | --- |
| *Actinobacteriota* | *Actinobacteria* | *Pseudonocardiales* | *Pseudonocardiaceae* | *Pseudonocardia* | 1.0 | 0.5 | 3.2 | 0.1 | 0.0 |
|  |  | *Corynebacteriales* | *Nocardiaceae* | *Nocardia* | 0.0 | 1.0 | 2.1 | 0.0 | 4.7 |
|  |  | *Streptomycetales* | *Streptomycetaceae* | *Streptomyces* | 0.0 | 0.0 | 1.1 | 0.0 | 12.8 |
|  |  | *Micrococcales* | *Promicromonosporaceae* | *Isoptericola* | 0.0 | 0.0 | 2.0 | 0.0 | 0.0 |
|  |  | *Streptosporangiales* | *Streptosporangiaceae* | *Nonomuraea* | 0.0 | 0.0 | 1.4 | 0.0 | 0.0 |
|  | *Thermoleophilia* | *Gaiellales* | *Uncultured* | uncultured | 0.6 | 0.2 | 1.5 | 0.0 | 0.0 |
| *Bacteroidota* | *Chitinophagia* | *Chitinophagales* | *Chitinophagaceae* | *Chitinophaga* | 0.0 | 0.0 | 3.2 | 0.0 | 0.0 |
|  |  |  |  | *Sediminibacterium* | 6.2 | 2.9 | 0.0 | 0.1 | 0.1 |
|  | *Flavobacteria* | *Flavobacteriales* | *Flavobacteriaceae* | *Salegentibacter* | 0.1 | 0.0 | 6.0 | 0.0 | 0.0 |
|  |  |  |  | *Flavobacterium* | 0.0 | 0.0 | 1.1 | 0.0 | 0.0 |
| *Firmicutes* | *Bacilli* | *Bacillales* | *Bacillaceae* | *Halalkalibacillus* | 0.0 | 0.0 | 2.2 | 0.0 | 0.0 |
|  |  |  |  | *Halobacillus* | 0.0 | 0.0 | 1.3 | 0.0 | 0.0 |
|  |  |  |  | *Bacillus* | 0.2 | 0.0 | 3.1 | 0.9 | 0.0 |
| *Nitrospirae* | *Nitrospira* | *Nitrospirales* | *Nitrospiraceae* | *Nitrospira* | 0.0 | 0.5 | 0.6 | 0.6 | 5.3 |
| *Proteobacteria* | *Betaproteobacteria* | *Burkholderiales* | *Burkholderiaceae* | *Limnobacter* | 1.8 | 25.8 | 0.0 | 0.0 | 8.7 |
|  |  |  | *Alcaligenaceae* | *Achromobacter* | 0.0 | 1.9 | 0.6 | 50.9 | 0.5 |
|  |  |  | *Comamonadaceae* | *Hydrogenophaga* | 1.3 | 0.7 | 1.4 | 0.1 | 0.8 |
|  |  |  |  | *__* | 2.4 | 0.4 | 0.1 | 0.1 | 0.2 |
|  |  | *Nitrosomonadales* | *Sterolibacteriaceae* | *Sulfuritalea* | 0.0 | 0.0 | 2.7 | 0.0 | 0.0 |
|  |  | *Rhodocyclales* | *Azonexaceae* | *Dechloromonas* | 0.0 | 0.0 | 2.6 | 0.0 | 0.0 |
|  | *Gammaproteobacteria* | *Alteromonadales* | *Alteromonadaceae* | *Marinobacter* | 0.4 | 0.0 | 12.5 | 0.0 | 0.0 |
|  |  |  | *Idiomarinaceae* | *Idiomarina* | 0.1 | 0.0 | 5.2 | 0.0 | 0.0 |
|  |  | *Pseudomonadales* | *Pseudomonadaceae* | *Rhizobacter* | 0.7 | 2.4 | 0.1 | 0.0 | 0.0 |
|  |  |  |  | *Pseudomonas* | 3.3 | 19.0 | 1.7 | 20.0 | 9.6 |
|  |  |  | *Moraxellaceae* | *Acinetobacter* | 1.0 | 0.2 | 0.0 | 1.3 | 0.8 |
|  |  | *Oceanospirillales* | *Halomonadaceae* | *Halomonas* | 0.0 | 0.0 | 1.6 | 0.0 | 0.0 |
|  |  | *Xanthomonadales* | *Xanthomonadaceae* | *Pseudoxanthomonas* | 0.1 | 2.8 | 3.3 | 0.1 | 5.8 |
|  |  |  |  | *Luteimonas* | 1.3 | 0.0 | 1.8 | 0.0 | 0.0 |
|  |  |  |  | *Stenotrophomonas* | 0.0 | 0.0 | 2.1 | 0.0 | 0.0 |
|  |  |  |  | *Lysobacter* | 0.1 | 0.3 | 3.1 | 0.0 | 0.2 |

| **Phylum** | **Class** | **Order** | **Family** | **Genus** | **BETH** | **CAS1** | **BEAR** | **IMM** | **MOU2** |
| --- | --- | --- | --- | --- | --- | --- | --- | --- | --- |
|  | *Alphaproteobacteria* | *Hyphomicrobiales* | *Rhizobiaceae* | *Allorhizobium-Neorhizobium-Pararhizobium-Rhizobium* | 1.5 | 6.4 | 1.5 | 7.9 | 4.1 |
|  |  |  |  | *__* | 1.4 | 0.9 | 0.3 | 0.1 | 2.5 |
|  |  |  |  | *Ensifer* | 0.0 | 10.8 | 0.1 | 0.1 | 0.6 |
|  |  |  | *Phyllobacteriaceae* | *Aliihoeflea* | 3.2 | 0.1 | 3.3 | 0.0 | 0.0 |
|  |  |  |  | *Aminobacter* | 0.0 | 0.1 | 0.0 | 1.1 | 0.4 |
|  |  |  |  | *Mesorhizobium* | 0.3 | 0.1 | 0.4 | 0.0 | 3.7 |
|  |  |  | *Hyphomicrobiaceae* | *Hyphomicrobium* | 15.1 | 3.3 | 0.2 | 3.0 | 3.3 |
|  |  |  | *Pseudoxanthobacteraceae* | *Pseudoxanthobacter* | 8.6 | 0.1 | 0.0 | 0.0 | 0.6 |
|  |  |  | *Xanthobacteraceae* | *__* | 0.5 | 1.1 | 0.0 | 0.5 | 3.6 |
|  |  |  | *Devosiaceae* | *Devosia* | 2.2 | 0.3 | 0.9 | 0.0 | 2.1 |
|  |  |  | *Boseaceae* | *Bosea* | 0.0 | 0.8 | 0.0 | 0.9 | 1.4 |
|  |  | *Caulobacterales* | *Caulobacteraceae* | *Brevundimonas* | 22.3 | 0.6 | 1.9 | 3.1 | 0.6 |
|  |  |  |  | *Caulobacter* | 0.4 | 4.5 | 1.3 | 0.5 | 8.5 |
|  |  | *Sphingomonadales* | *Sphingomonadaceae* | *Sphingopyxis* | 10.8 | 1.8 | 0.6 | 0.1 | 0.8 |
|  |  |  |  | *Altererythrobacter* | 0.4 | 0.0 | 1.6 | 0.0 | 0.0 |
|  |  | *Rhodospirillales* | *Reyranellaceae* | *Reyranella* | 0.2 | 2.1 | 0.0 | 1.2 | 2.1 |
|  |  |  | *Thalassobaculaceae* | *Thalassobaculum* | 1.1 | 0.0 | 0.0 | 0.0 | 0.0 |
| *Verrucomicrobia* | *Opitutae* | *Opitutales* | *Opitutaceae* | *Lacunisphaera* | 0.0 | 0.1 | 0.0 | 0.0 | 1.4 |
| WPS-2 | WPS-2 | WPS-2 | WPS-2 | WPS-2 | 1.5 | 0.0 | 0.0 | 0.5 | 0.0 |

**Table S6.** Bacterial genera identified in the precipitates of drip waters from Nerja Cave that have been reported as involved in calcite precipitation processes.

| **Genus** | **References** |
| --- | --- |
| *Achromobacter* | Busquets et al. (2014) |
| *Acinetobacter* | Ferrer et al. (1988), Banerjee & Joshi (2014), Busquets et al. (2014) |
| *Bacillus* | Sanchez-Moral et al. (2004), Sprocati et al. (2008), Achal & Pan (2011), Chu et al. (2012), Rivadeneyra Torres et al. (2013), Wei et al. (2015), Seifan et al. (2016), Meier et al. (2017), Castro-Alonso et al. (2019), Oualha et al. (2020), Omoregie et al. (2021), Rajasekar et al. (2021) |
| *Brevundimonas* | Busquets et al. (2014), Wei et al. (2015), Khan et al. (2021) |
| *Caulobacter* | Rivadeneyra Torres et al. (2013) |
| *Ensifer* | Hatayama & Saito (2019), Hatayama (2020) |
| *Flavobacterium* | Ferrer et al. (1988), Meier et al. (2017) |
| *Halomonas* | Rothenstein et al. (2012) |
| *Hydrogenophaga* | Busquets et al. (2014) |
| *Idiomarina* | Gónzalez-Muñoz et al. (2008), Oliveira et al. (2014) |
| *Lysobacter* | Sanchez-Moral et al. (2004), Sprocati et al. (2008) |
| *Marinobacter* | Sánchez-Román et al. (2007), Khansha et al. (2017) |
| *Mesorhizobium* | Tan et al. (2018) |
| *Nocardia* | Ercole et al. (2012) |
| *Pseudomonas* | Sprocati et al. (2008), Rusznyák et al. (2012), Busquets et al. (2014), Baskar et al. (2016), Meier et al. (2017), Castro-Alonso et al. (2019), Hatayama (2020), Rajasekar et al. (2021) |
| *Rhizobium* | Meier et al. (2017) |
| *Sphingopyxis* | Meier et al. (2017), Rajasekar (2018) |
| *Stenotrophomonas* | Sanchez-Moral et al. (2004), Sprocati et al. (2008), Rusznyák et al. (2012), Park et al. (2013), Busquets et al., (2014) |
| *Streptomyces* | Groth et al., (2001), Sprocati et al. (2008), Busquets et al. (2014), Baskar et al. (2016), Cao et al. (2016), Maciejewska et al. (2017), Meier et al. (2017), Eltarahony et al. (2021) |

**References**

Achal V, Pan X. 2011. Characterization of urease and carbonic anhydrase producing bacteria and their role in calcite precipitation. *Curr. Microbiol.* 62: 894–902.

Banerjee S, Joshi SR. 2016. Culturable bacteria associated with the caves of Meghalaya in India contribute to speleogenesis. *J. Cave Karst Stud.* 78: 144–157.

Baskar S, Routh J, Baskar R, Kumar A, Miettinen H, Itävaara M. 2016. Evidences for microbial precipitation of calcite in speleothems from Krem Syndai in Jaintia Hills, Meghalaya, India, *Geomicrobiol. J.* 33: 906–933.

Busquets A, Fornós JJ, Zafra F, Lalucat J, Merino A. 2014. Microbial communities in a coastal cave: Cova des Pas de Vallgornera (Mallorca, Western Mediterranean). *Int. J. Speleol.* 43: 205–216.

Cao C, Jiang J, Sun H, Huang Y, Tao F, Lian B. 2016. Carbonate mineral formation under the influence of limestone-colonizing actinobacteria: Morphology and polymorphism. *Front. Microbiol.*  7: 366.

Carrasco Cantos, F, Liñán Baena C, Durán Valsero JJ, Andreo Navarro B, Vadillo Pérez I. 2002. Modificaciones de los parámetros ambientales de la Cueva de Nerja provocadas por la entrada de visitantes. *Geogaceta* 31: 15–18.

Castro-Alonso MJ, Montañez-Hernandez LE, Sanchez-Muñoz MA, Macias Franco MR, Narayanasamy R, Balagurusamy N. 2019. Microbially induced calcium carbonate precipitation (MICP) and its potential in bioconcrete: Microbiological and molecular concepts. *Front. Mater.* 6: 126.

Chu J, Stabnikov V, Ivanov V. 2012. Microbially induced calcium carbonate precipitation on surface or in the bulk of soil. *Geomicrobiol. J.* 29: 544–549.

Eltarahony M, Zaki S, Kamal A, Abd-El-Haleem D. 2021. Calcite and vaterite biosynthesis by nitrate dissimilating bacteria in carbonatogenesis process under aerobic and anaerobic conditions. *Geomicrobiol. J.* 38: 791–808.

Ercole C, Bozzelli P, Altieri F, Cacchio P, Del Gallo M. 2012. Calcium carbonate mineralization: Involvement of extracellular polymeric materials isolated from calcifying bacteria. *Microsc. Microanal.* 18: 829–839.

Ferrer MR, Quevedo-Sarmiento J, Rivadeneyra MA, Bejar V, Delgado R, Ramos-Cormenzana A. 1988. Calcium carbonate precipitation by two groups of moderately halophilic microorganisms at different temperatures and salt concentrations. *Curr. Microbiol.* 17: 22–227.

González-Muñoz MT, De Linares C, Martínez-Ruiz F, Morcillo F, Martín-Ramos D, Arias JM. 2008. Ca–Mg kutnahorite and struvite production by *Idiomarina* strains at modern seawater salinities. *Chemosphere* 72: 465–472.

Groth I, Schumann P, Laiz L, Sanchez-Moral S, Cañaveras JC, Saiz-Jimenez C. 2001. Geomicrobiological study of the Grotta dei Cervi, Porto Badisco, Italy. *Geomicrobiol. J.* 18: 241–258.

Hatayama K. 2020. Manganese carbonate precipitation induced by calcite-forming bacteria. *Geomicrobiol. J.* 37: 603-609.

Hatayama K, Saito K. 2019. Calcite formation induced by *Ensifer adhaerens, Microbacterium testaceum, Paeniglutamicibacter kerguelensis, Pseudomonas protegens* and *Rheinheimera texasensis*. *Anton. Leeuw*. 112: 711–721.

Jiménez de Cisneros C, Peña A, Caballero E, Liñán C. 2020. A multiparametric approach for evaluating the current carbonate precipitation and external soil of Nerja Cave (Málaga, Spain). *Int. J. Environ. Res.* 15: 1–13.

Khan I, Rafiq M, Zada S, Jamil SUU, Hasan F. 2021. Calcium carbonate precipitation by rock dwelling bacteria in Murree Hills, Lower Himalaya Range Pakistan. *Geomicrobiol. J.* 38: 231-236.

Khansha J, Ranjbaran M, Amoozegar MA. 2017. Isolation and identification of halophilic and halotolerant bacteria from Badab-e Surt Travertine Spring, Kiasar, Iran, and investigation of calcite biomineralization induction. *Geomicrobiol. J.* 35: 64–73.

Liñán C, Carrasco F, Vadillo I, Garrido A. 2008. Estudios hidrogeológicos en la Cueva de Nerja. In: López-Geta JA, Rubio Campos JC, Martín-Machuca M, eds. Agua y Cultura, VII Simposio del Agua en Andalucía. Madrid: IGME, vol 2, 673-683.

Maciejewska M, Adam D, Naômé A, Martinet L, Tenconi E, Całusinska M, Delfosse P, Hanikenne M, Baurain D, Compère P, Carnol M, Barton HA, Rigali S. 2017. Assessment of the potential role of *Streptomyces* in cave moonmilk formation. *Front. Microbiol.* 8: 1181.

Meier A, Kastner A, Harries D, Wierzbicka-Wieczorek M, Majzlan J, Büchel G, Kothe E. 2017. Calcium carbonates: Induced biomineralization vs. controlled macromorphology? *Biogeosciences* 14: 4867–4878.

Oliveira PJV, da Costa MS, Costa JNP, Nobre MF. 2015. Comparison of the ability of two bacteria to improve the behavior of sandy soil. *J. Mat. Civil Eng.* 27: 1.

Omoregie AI, Palombo EA, Nissom PM. 2021. Bioprecipitation of calcium carbonate mediated by ureolysis: A review. *Environ. Eng. Res.* 26, 200379.

Oualha M, Bibi S, Sulaiman M, Zouari N. 2020. Microbially induced calcite precipitation in calcareous soils by endogenous *Bacillus cereus*, at high pH and harsh weather. *J. Environ. Manag.* 257: 109965.

Park J-M, Park S-J, Ghim S-Y. 2013. Characterization of three antifungal calcite-forming bacteria, *Arthrobacter nicotianae* KNUC2100, *Bacillus thuringiensis* KNUC2103, and *Stenotrophomonas maltophilia* KNUC2106, derived from the Korean Islands, Dokdo and their application on mortar. *J. Microbiol. Biotechnol.* 23: 1269–1278.

Rajasekar A. 2018. Calcite-precipitating indigenous bacteria in landfills and their application towards ground improvement. Ph D. Thesis. University of Liverpool.

Rajasekar A, Moy CKS, Wilkinson S, Sekar R. 2021. Microbially induced calcite precipitation performance of multiple landfill indigenous bacteria compared to a commercially available bacteria in porous media. *PLoS ONE* 16: e0254676.

Rivadeneyra Torres A, Martinez-Toledo M., Gonzalez-Martinez A, Gonzalez-Lopez J, Martin-Ramos D, Rivadeneyra MA. 2013. Precipitation of carbonates by bacteria isolated from wastewater samples collected in a conventional wastewater treatment plant. *Int. J. Sci. Technol.* 10: 141–150.

Rothenstein D, Baier J, Schreiber TD, Barucha V, Bill J. 2012. Influence of zinc on the calcium carbonate biomineralization of *Halomonas halophila*. *Aquat. Biosyst*. 8: 31.

Rusznyák A, Akob DM, Nietzsche S, Eusterhues K, Totsche KU, Neu TR, Frosch T, Popp J, Keiner R, Geletneky J, Katzschmann L, Schulze E-D, Küsel K. 2012. Calcite biomineralization by bacterial isolates from the recently discovered pristine karstic Herrenberg Cave. *Appl. Environ. Microbiol.* 78: 1157–1167.

Sanchez-Moral S, Luque L, Cañaveras JC, Laiz L, Jurado V, Saiz-Jimenez C. 2004. Bioinduced barium precipitation in St Callixtus and Domitilla catacombs. *Ann. Microbiol.* 54: 1–12.

Sánchez-Román M, Rivadeneyra MA, Vasconcelos C, McKenzie JA. 2007. Biomineralization of carbonate and phosphate by moderately halophilic bacteria. *FEMS Microbiol. Ecol.* 61, 273–284.

Seifan M, Samani AK, Berenjian A. 2016. Induced calcium carbonate precipitation using *Bacillus* species. *Appl. Microbiol. Biotechnol.* 100: 9895–9906.

Sprocati AR, Alisi C, Tasso F, Vedovato E, Barbabietola N, Cremisini C. 2008. A microbiological survey of the Etruscan Mercareccia Tomb (Italy): Contribution of microorganisms to deterioration and restoration. In: 9th International Conference on NDT of Art, Jerusalem Israel, 25-30 May 2008.

Tan SI, Han YL, Yu YJ, Chiu CY, Chang YK, Ouyang S, Fan KC, Lo KH, Ng IS. 2018. Efficient carbon dioxide sequestration by using recombinant carbonic anhydrase. *Process Biochem.* 73: 38–46.

Wei S, Cui H, Jiang Z, Liu H, He H, Fang N. 2015. Biomineralization processes of calcite induced by bacteria isolated from marine sediments. *Braz. J. Microbiol.* 46: 455–464.
